# A Single Bivalent Efficiently Inhibits Cyclin B1 Degradation and Polar Body Extrusion in Mouse Oocytes Indicating Robust SAC during Female Meiosis I

**DOI:** 10.1371/journal.pone.0027143

**Published:** 2011-11-18

**Authors:** Steffen Hoffmann, Bernard Maro, Jacek Z. Kubiak, Zbigniew Polanski

**Affiliations:** 1 Department of Developmental Biology, Max Planck Institute of Immunobiology, Freiburg, Germany; 2 UMR 6061 CNRS/ University of Rennes 1, Mitosis & Meiosis Group, IFR 140 GFAS, Rennes, France; 3 Department of Genetics and Evolution, Institute of Zoology, Jagiellonian University, Cracow, Poland; Institut de Génétique et Développement de Rennes, France

## Abstract

The Spindle Assembly Checkpoint (SAC) inhibits anaphase until microtubule-to-kinetochore attachments are formed, thus securing correct chromosome separation and preventing aneuploidy. Whereas in mitosis even a single unattached chromosome keeps the SAC active, the high incidence of aneuploidy related to maternal meiotic errors raises a concern about the lower efficiency of SAC in oocytes. Recently it was suggested that in mouse oocytes, contrary to somatic cells, not a single chromosome but a critical mass of chromosomes triggers efficient SAC pointing to the necessity of evaluating the robustness of SAC in oocytes. Two types of errors in chromosome segregation upon meiosis I related to SAC were envisaged: (1) SAC escape, when kinetochores emit SAC-activating signal unable to stop anaphase I; and (2) SAC deceive, when kinetochores do not emit the signal. Using micromanipulations and live imaging of the first polar body extrusion, as well as the dynamics of cyclin B1 degradation, here we show that in mouse oocytes a single bivalent keeps the SAC active. This is the first direct evaluation of SAC efficiency in mouse oocytes, which provides strong evidence that the robustness of SAC in mammalian oocytes is comparable to other cell types. Our data do not contradict the hypothesis of the critical mass of chromosomes necessary for SAC activation, but suggest that the same rule may govern SAC activity also in other cell types. We postulate that the innate susceptibility of oocytes to errors in chromosome segregation during the first meiotic division may not be caused by lower efficiency of SAC itself, but could be linked to high critical chromosome mass necessary to keep SAC active in oocyte of large size.

## Introduction

Embryonic aneuploidy is the major source of pregnancy loss or developmental disorders such as Down's Syndrome [1]. Cells, including oocytes, reduce the risk of aneuploidy through the function of the Spindle Assembly Checkpoint (SAC) which inhibits the onset of anaphase until microtubule-to-kinetochore attachments are formed [2]–[5]. SAC controls the timing of degradation of at least two proteins: securin responsible for maintaining chromosome cohesion, and cyclin B1 necessary to keep the cell in M-phase. The absence of microtubule-to-kinetochore attachments keeps the SAC active enabling it to generate a signal inhibiting the Anaphase Promoting Complex/Cyclosome (APC/C) which is the key component of securin/cyclin B degradation pathway. Once the chromosomes achieve bi-orientation through establishment of microtubule-to-kinetochore attachments SAC is inactivated and the inhibitory signal exerted on APC/C ceases [5]. This in turn leads to simultaneous degradation of securin and cyclin B driving the cell into anaphase and coordinated M-phase exit. In this way SAC delays chromosome separation until they achieve proper orientation ensuring their faithful segregation. Interestingly, in somatic cells, cyclin B1 also controls chromosome cohesion via inhibition of separase, a cystein protease that cleaves cohesins [6]. However, this role of cyclin B1 seems minor in mouse oocytes at least during second metaphase stage [7] suggesting important differences in hierarchy of mechanisms controlling chromosome cohesion/separation in somatic and germ cells.

The functionality of SAC during mammalian female meiosis was reported by several groups [8]–[11]. However, the high incidence of aneuploidy related to maternal meiotic errors, especially occurring at meiosis I [12] raises a concern about the possible lower efficiency of SAC in oocytes [3] compared to mitotic cells in which even a single chromosome lacking microtubule-to-kinetochore attachments keeps the SAC active [13], [14]. Recently, Nagaoka and collaborators [4] suggested that the anaphase I onset in mouse oocytes requires stable bipolar attachment of a critical mass-but not all-of chromosomes. This conclusion strongly suggested differences in SAC functioning in oocytes *vs.* somatic cells. Also the quoted above differences in the role of cyclin B1 in the control of chromosome cohesion/separation in somatic cells [6] *vs.* oocytes [7] strengthen the need to reevaluate the SAC function in oocytes. In this paper we show that at first meiotic division a single bivalent delays anaphase onset in a SAC-dependent manner in oocytes with cytoplasm volume reduced to half. This shows efficient function of SAC during first meiosis in mouse oocytes, and permits to evaluate the critical mass of chromosomes activating SAC in these cells.

## Results

### Cyclin B1 is precociously degraded in achromosomal oocyte halves

**Figure 1:**
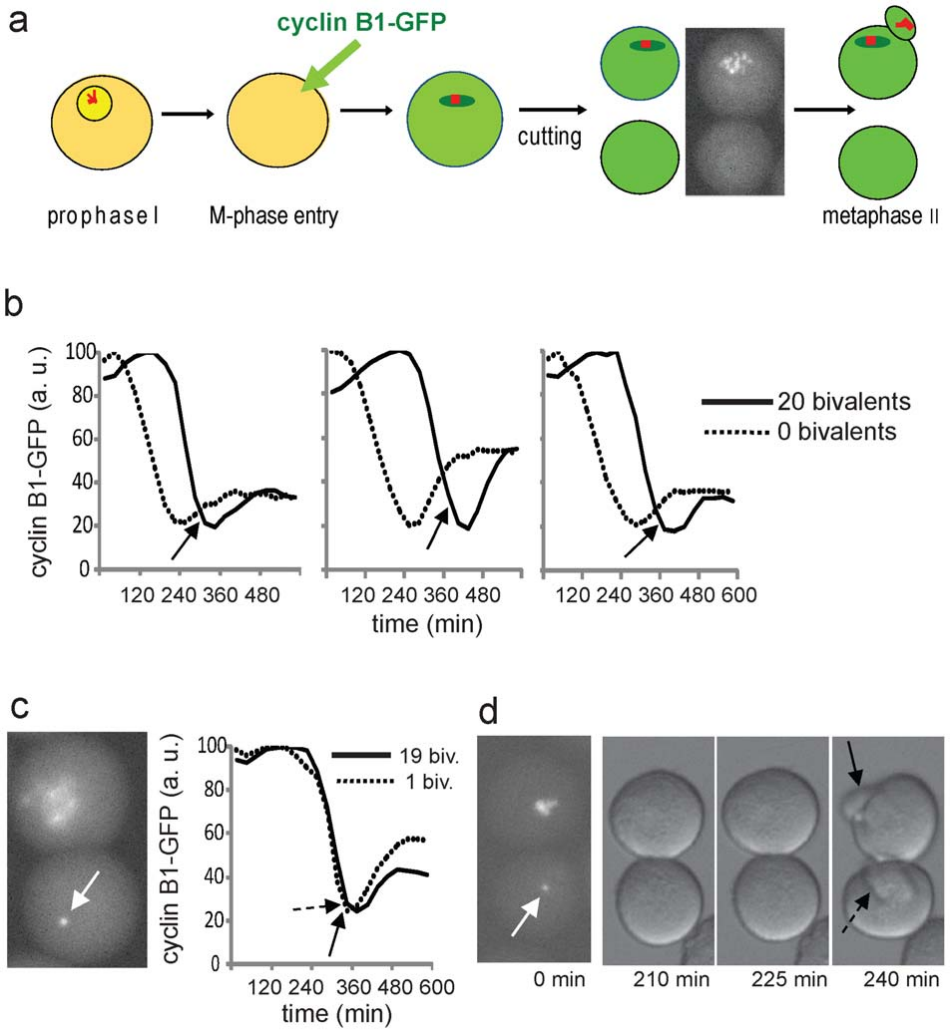
The lack of chromosomes results in precocious degradation of cyclin B1, while the presence of a single bivalent rescues the normal timing of degradation. (a) The design of experiment: oocytes were injected with cyclin B-GFP RNA at the onset of meiotic maturation and cut into two halves containing separately the whole set of chromosomes and cytoplasm only (vital Hoechst staining of chromatin in such an pair is shown). (b) Cyclin B1-GFP degradation in the pair (leftmost) shown in A and two other sister pairs (solid line - half with all chromosomes; dotted line – half with cytoplasm only). In all three cases the degradation of cyclin B1-GFP in the cytoplasmic half precedes the degradation in its sister half containing chromosomes for 2–3 hours. Solid arrows indicate the time of extrusion of the first polar body in chromosomes-containing halves. (c) Live Hoechst image of the sister pair of halves where cutting produced one half containing a single bivalent (white arrow) whereas the other half contained the rest of the chromosomes (slightly out of focus) and corresponding profile of cyclin B1-GFP in this pair showing the synchrony in degradation in the half containing one bivalent and the sister half containing the rest of chromosomes. The first polar bodies in both halves were also extruded synchronously (dotted arrow and solid arrow for the half containing the single bivalent and the sister half with the rest of chromosomes, respectively). (b) and (c) show data of a single experiment in which four pairs of halves were imaged. (d) A sister pair of halves where one half contains a single bivalent (white arrow) and the other half contains the rest of chromosomes. This pair was not subjected to cyclin B1-GFP imaging, however, the bright field DIC video microscopy showed that the extrusion of the first polar body (an equivalent of cytokinesis in oocytes) occurred at the same time in the half containing a single bivalent (dotted arrow) and in the half containing the rest of chromosomes (solid arrow).

Using live imaging of GFP-tagged cyclin B1 we have tested the dynamics of cyclin B1 degradation in oocytes which is the reliable tool to study the SAC activity [8], [15], [16]. Oocytes expressing cyclin B1-GFP were bisected to produce couplet (pair) of two halves originating from the same oocyte, one containing and the other lacking chromosomes (Fig. 1a). The absence of chromosomes (Fig. 1b, inset showing vital Hoechst staining) along with kinetochores, which are the primary sites generating the SAC inhibitory signal, means that the SAC cannot function. Accordingly, in all cases (51 pairs analyzed) the degradation of cyclin B1-GFP in the oocyte half devoid of chromosomes clearly preceded its degradation in the sister half containing chromosomes (Fig. 1b), since only in the latter the SAC inhibits degradation machinery until establishment of bi-orientation. The onset of cyclin B1-GFP degradation in the chromosome-containing half was always delayed in relation to the chromosome-devoid sister half with minimum delay between halves (observed in 4 couplets; 8%) amounting to 30 minutes. Thus, in 92% of the couplets consisting of achromosomal and chromosome-containing halves the chromosomal half was delayed in the onset of cyclin B1 degradation for more than 30 minutes. On average, for the 51 analyzed couplets the chromosome-containing karyoplast started to degrade cyclin B1-GFP 129 minutes after the achromosomal cytoplast. This is consistent with previous reports showing that disruption of the SAC in mouse oocytes accelerates anaphase and cytokinesis by 2–4 hours [8], [9], [17].

### Cyclin B1 is synchronously degraded in whole oocytes and oocyte halves with a single bivalent

**Figure 2:**
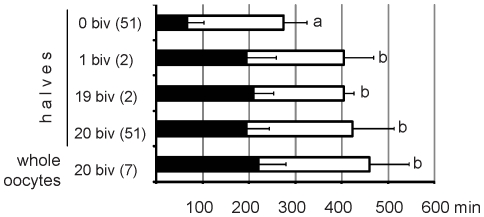
Timing of cyclin B1-GFP degradation during meiotic maturation of oocyte halves containing different number of chromosomes. The black part of the bars represent the mean time of start of cyclin B1-GFP degradation (± SD), the white part of bars represent the mean time elapsing from the start of degradation to the end of degradation (the time point at which the signal intensity drops to minimum; ± SD). The number of oocytes/oocyte halves analysed is given in parentheses. Different letters indicate statistically significant differences (ANOVA with Tukey HSD post hoc test, P<0.01 for all indicated differences).

In three cases, oocyte bisection resulted in generation of one half containing only one bivalent and the other one containing the remaining 19 bivalents (Fig. 1c, d, Hoechst staining). Two such couplets were subjected to imaging. In the first case degradation of the cyclin B1-GFP in the half containing one bivalent and in the half containing 19 bivalents occurred exactly at the same time and with the same dynamics (Fig. 1c). In the second case the degradation of the cyclin B1-GFP in the half containing one bivalent started only 30 minutes earlier than in the half containing 19 bivalents. The third couplet was not subjected to cyclin B1-GFP imaging, however bright field DIC video microscopy showed that cytokinesis occurred at the same time in both sister halves (Fig. 1d). Thus, in each case when a couplet consisted of achromosomal and chromosome-containing half the cyclin B1-GFP degradation occurred earlier in achromosomal half. By contrast, among three couplets in which one half contained only single bivalent and the second half contained the rest 19 bivalents, in two couplets the cyclin B1-GFP degradation or cytokinesis occurred at the same time in both halves, and in the third couplet only 30 minutes earlier in single-bivalent-containing half. Table 1 shows such direct comparison of the timing of cyclin B1-GFP degradation or cytokinesis between sister halves for each couplet. The data are arranged in order to apply Fisher Exact Probability Test (statistical test which is specifically suited for small quantities) which showed that the difference in the pattern of timing of cyclin B1-GFP degradation between 0/20 bivalents couplets and 1/19 bivalents couplet is highly statistically significant (Table 1). Thus, a single bivalent is able to inhibit anaphase signaling in a manner similar to 19 bivalents. We also present the real timing of cyclin B1-GFP degradation for oocyte halves containing 0, 1, 19 and 20 bivalents as well as for the intact (non-bisected) whole oocytes (Fig. 2). On average the cyclin B1-GFP degradation in the absence of any bivalent started after 66 minutes of the culture, thus significantly earlier (p<0.01, ANOVA) than in all other groups containing different numbers of bivalents (Fig. 2). On the other hand, whole oocytes or oocyte halves containing 1, 19 or 20 bivalents did not differ significantly in the average timing of the onset of cyclin B1-GFP degradation (range 195 to 220 minutes; Fig. 2). This analysis shows that the presence of only one bivalent sets up the normal timing of cyclin B1 degradation typical for the intact (non-bisected) oocytes. Thus, two statistical tests in a different and independent way prove that the oocyte halves containing a single bivalent behave similarly to the oocyte halves with 19 or 20 bivalents, but in a totally different way than the oocyte halves devoid of all chromosomes in which cyclin B degradation occurs prematurely.

**Table 1:** Comparison of timing of cyclin B1-GFP degradation or cytokinesis within couplets of oocyte halves.

No. of bivalents in sister halves	Onset of cyclin B1-GFP degradation or cytokinesis	
	earlier in 0 (or 1 bivalent) half	similar in both halves#
0/20^a^	47	4
1/19^a^	0	2(3)^b^

# similar means that the difference between halves within the couplet is not higher than 30 minutes (in fact in 1/19 pairs the timing is the same in two cases and only in the third one differs by 30 minutes).

a the difference in pattern of cyclin B1-GFP degradation between two types of couplets (0/20 and 1/19) is statistically significant (47 vs 4 and 0 vs 2; p<0.02, Fisher Exact Probability Test).

b Assuming that cytokinesis reflects the timing of cyclin B1 degradation as shown elsewhere one may add one 1/19 couplet for which only cytokinesis data are available (Fig. 1d). In such case the significance of difference in pattern of cyclin B1-GFP degradation/cytokinesis between two types of couplets reaches p<0.002 (47 vs 4 and 0 vs 3; Fisher Exact Probability Test).

### Cyclin B1 dynamics in oocytes halves are regulated by SAC

**Figure 3:**
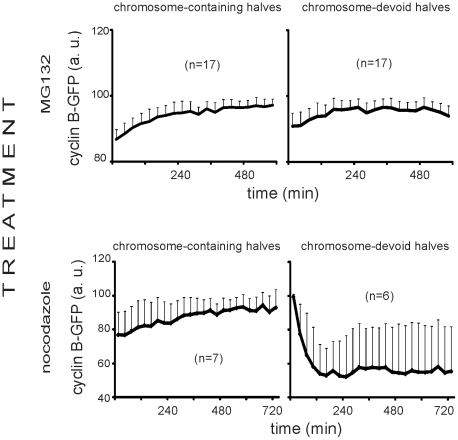
Cyclin B1-GFP behaviour in oocyte halves cultured in the presence of proteosome inhibitor MG132 or nocodazole. Data represent mean ± SD. The number of ooocyte halves analysed is given in parentheses.

To make sure that the precocious degradation of cyclin B1-GFP in the absence of chromosomes results from silencing of the SAC, we performed imaging of oocyte halves in the conditions which normally prevent cyclin B1 degradation. The proteosome inhibitor MG132 acts downstream of the SAC and inhibits cyclin B1 degradation in oocytes and even in the absence of active SAC [18]. Accordingly, under the influence of MG132 the level of cyclin B1-GFP remained high and stable in both chromosome-containing as well as chromosome-lacking halves throughout the whole imaging period (Fig. 3). On the other hand, nocodazole, an inhibitor of microtubule assembly, removes kinetochore-microtubule interactions and prevents chromosome bi-orientation, thus keeping the SAC active [9]. The presence of nocodazole indeed prevented cyclin B1-GFP degradation in the chromosome-containing halves which is characteristic also for whole oocytes upon microtubule disassembly [19]. By contrast, in the chromosome-devoid halves subjected to nocodazole treatment cyclin B1-GFP degradation occurred unaffected (Fig. 3). These results agree perfectly with the mechanism of SAC action: in the absence of kinetochores (essential for SAC activity) the SAC is off and accordingly nocodazole cannot prevent cyclin B1-GFP degradation. Thus, the absence of chromosomes makes SAC inactive and results in precocious degradation of cyclin B1, however even a single bivalent restores the full SAC activity for a period necessary for its correct orientation.

### Dynamics of cyclin B1 degradation differ in presence and absence of SAC

Quantification of the intensity of the cyclin B1-GFP signal enabled as to determine the onset of cyclin B1-GFP degradation as well as the time when the signal drops to minimum, which we consider as the end of degradation. This allows to calculate the duration of degradation as well as the relative (based on the signal intensity) amount of cyclin B1-GFP degraded in the achromosomal or chromosome containing oocyte halves (Table 2). Such calculations show that the efficiency of cyclin B1-GFP degradation is significantly higher in the presence of chromosomes, which is somehow consistent with previous studies demonstrating that proteolysis of cyclin B requires the intact spindle [19]. Although impaired, the cyclin B1-GFP degradation in achromosmal halves occurs, however, quite efficiently (Table 2, also Fig. 1). Our previous study showed that the microtubules in achromosomal fragments of oocytes are able to form polarized structures including perfectly formed achromosomal bipolar spindles [20]. It is possible that cyclin B1-GFP degradation in achromosomal halves of oocytes can depend on such structures.

**Table 2:** Evaluation of the APC activity during ongoing cyclin B1-GFP degradation in oocyte halves containing or devoid of chromosomes as well as in whole oocytes.

Chromosomes	Characteristics of cyclin B1-GFP degradation				APC activity (extent/duration)^4^
	Duration (hours)^1^	Signal intensity (A. U.) at		Extent (signal at start – signal at end)^3^	
		start of degradation^2^	end of degradation		
present	**3.8±1.3**	**100** *	**30.7±11.0**	**69.3±11.0** #	**18.2±2.9** ¶
absent	**3.5±1.0**	**85.3±11.2** *	**30.1±10.9**	**55.2±11.9** # &	**15.9±3.4** ¶ $
present (whole oocytes)	**4.0±0.0**	**100**	**24.5±7.4**	**75.5±7.4** &	**19.7±5.2** $

1 time elapsing between the beginning and the end of degradation.

2 for each couplet of halves the data were normalized by setting the signal for chromosomal half at the beginning of degradation as 100.

3 the signal value at the end of degradation subtracted from signal value at the beginning of degradation gives the relative amount of cyclin B1-GFP which was degraded and thus defining the extent of degradation.

4 Extent of degradation divided by the duration of degradation gives the average efficiency of cyclin B1-GFP degradation.

Data shown as Mean ± SD.

The values marked with the same superscript differ significantly.

*, ^#^P<0.0001;

¶ P<0.0005,

& P<0.0001,

$ P<0.01 (repeated T test).

## Discussion

Our analyses of cyclin B1 dynamics and timing the first polar body extrusion in whole and bisected oocytes demonstrate that SAC is robust during the first meiotic division in mouse oocytes and that a single bivalent is sufficient to keep SAC active for a prolonged time. This first evaluation of the efficiency of SAC in mammalian oocytes shed new light on SAC function in female meiosis.

Nagaoka and collaborators [4] have shown that the anaphase I onset in mouse oocytes depends on attachment of a critical mass of chromosomes and not of all of hem. This could argue for a fundamental difference in the strength of the SAC-activating signal in oocytes and somatic cells. The evaluation of the robustness of SAC and the nature of eventual differences in functioning of SAC in oocytes and somatic cells was therefore a key point in understanding the origin of the meiotic aneuploidy leading to developmental disorders like Down's syndrome in humans ([17], this study). Our current analyses clearly show that in mouse oocytes a single bivalent generates a robust signal supporting SAC activity for several hours, thus for a period usually required for the proper orientation of chromosomes [8], [9]. This demonstrates that the effectiveness of the meiotic SAC in oocytes is comparable, but not necessarily identical with that of the mitotic SAC, where a single unattached chromosome keeps SAC active and delays anaphase [13], [14]. However, a single bivalent harbors four kinetochores in contrast to a mitotic chromosome having two. Thus, the SAC-activating signal generated by a bivalent is likely stronger than by a mitotic chromosome. Nagaoka and colleagues [4] show in oocytes of *Mlh1* mutant mice (independently by cytogenetics and misaligned chromosome counting) that on average 4 univalents possessing 8 kinetochores constitute the critical mass allowing SAC escape during anaphase I despite evidences that these kinetochores emit SAC-activating signal. Our results show that the equivalent number of kinetochores, i.e. 4/oocyte half, thus likely 8/whole oocyte, is sufficient to keep SAC active. Thus, the two reports allow drawing a very similar conclusion that 8 kinetochores *per* oocyte emit a signal of a critical value. Paradoxically, the correctness of the critical mass hypothesis does not exclude that SAC in oocytes differ from SAC in somatic cells. It rather suggests that in somatic cells 2 kinetochores emit the critical SAC-activating signal [13], [14] and in mouse oocyte 8 kinetochores could do so. Where does this difference come from? It is likely to be related to the cell size. The huge *Xenopus laevis* oocyte is probably the most sticking example: it has the functional SAC molecular machinery, but non-functional SAC and the most plausible explanation for this is related to the size of this oocyte where the activating signal dilutes in a large volume of cytoplasm as shown by studies of both cell extracts studies and manipulated oocytes [21], [22]. Thus, errors *via* SAC escape in mammalian oocytes are also likely related to their size.

Errors in chromosome separation during the first meiotic division in oocytes, being the major source of human aneuploidy, may also origin from SAC deceiving. Oocytes from X0 mice, which have univalent chromosome X next to remaining 19 bivalents did not show any delay/block in MI/MII transition suggesting that the SAC in oocytes is unable to detect such a single univalent [23]. Kouznetsova and colleagues [24] showed that univalents at female meiosis I undergo biorientation in a way typical for chromosomes during normal mitosis or second meiotic division. Apparently, such configuration, which satisfies SAC in mitosis and second meiosis, is recognized by SAC as the correct one also during the first meiosis. These results [24], in conjunction with our data and those by Nagaoka and collaborators [4], show that the elevated frequency of chromosome mis-segregation during the first meiotic division in mammalian oocytes does not result from a low efficiency of the SAC *per se*. It rather points out to involvement of a specific architecture of meiotic chromosomes increasing the incidence of SAC deceiving and the relationship between the critical mass of chromosomes/critical signal emitted by kinetochores necessary to efficiently activate SAC and the cell size. This hypothesis was recently reinforced by detailed analysis of metaphase spindle formation and bivalents congression to the metaphase plate in mouse oocytes by the group of Jan Ellenberg [25] precising our early observations and experiments [26]. Most importantly these papers show an important asynchrony between bivalents congression and the meiotic spindle formation resulting in numerous initial attachments of kinetochores with microtubules, which are routinely corrected via Aurora B/C kinase action. Such a mode of physiological spindle-kinetochore interactions may facilitate errors. Moreover, the increase of errors in chromosome segregation during meiosis one is also related to the maternal age [27]–[29]. The analyses by the group of Jan Ellenberg were conducted entirely with oocytes of young 8-week-old mice [25A]. It would be of great interest to compare in the future such analysis done with oocytes from old females in parallel with measuring SAC, as we show in our current paper. It could allow evaluating whether SAC regulation is involved in these age-related errors including Down's syndrome.

## Materials and Methods

### Ethics statement

Ethical approval for presented study was obtained from the Regierungspraesidium Freiburg under the permission entitled “Analyse der Entwisklungskapazitat des Maus-Oozyten nach Kern- oder Cytoplasma-Injektion oder Transfer” (No. T-01/56).

### Oocyte culture

OF1 mouse oocytes at the prophase stage of the first meiotic cycle were isolated from ovaries of adult mice into the M2 medium (Sigma Aldrich). The same medium was used for further culture/processing of oocytes and halves. Culture was performed at 37°C, whereas injection and oocyte cutting (bisection) was done at room temperature.

### Cyclin B1-GFP RNA injection, oocyte bisection and microscopic observations

After two hours of culture the oocytes which entered the first meiotic M-phase (indicated by disappearance of nuclear envelope) were injected with cyclin B1-GFP RNA [30]. After additional one hour of culture the zona pellucida was removed from injected oocytes by 5 minutes 0.25% pronase (B grade, Calbiochem) treatment in M2 medium. Following zona pellucida digestion oocytes were immediately bisected into two separate halves by glass needle on the soft agar surface into two halves as described previously [31]–[32]. Sister halves originated from the same oocyte were then in most experiments agglutinated for 5–10 minutes in the medium containing 400 µg/ml lectin (PHA-M, Sigma Aldrich), to maintain the two sisters together (which facilitated further analysis), followed by 30 minutes culture in M2 with vital Hoechst (bisbenzimide, H 33258, Sigma Aldrich, 1 µg/ml). Halves pairs were then cultured at 37°C on the stage of inverted Zeiss Axiovert 200 M microscope, equipped with fluorescence and DIC optics. Nocodazole and MG132 were used at concentrations 2 µg/ml and 20 µM, respectively. Immediately after placing the dishes with oocyte halves an image using filter appropriate for vital Hoechst was captured to allow visualization of chromatin. During culture images were captured every 15 minutes at GFP and DIC channels.

### Data treatment

The intensity of the fluorescence generated by GFP was measured on the acquired images using Adobe Photoshop. In each image analysed the virtual circular frame with the diameter slightly lees than the diameter of the oocyte half was created. This frame was superimposed over the oocyte half and the mean luminosity of the marked area was measured using «histogram» function. Then, the same frame was moved to cover the next oocyte half followed by measurement. Using the same frame all oocyte halves on all images within a whole single experiment were analysed. The same frame was also superimposed over the area where no oocyte halves were present (always the left lower corner of the image) to measure the background on each image. The background value was then subtracted from value obtained for each oocyte half analysed on a given image.
